# Female germline stem cells: recent advances, opportunities, and challenges to overcome

**DOI:** 10.1186/s13619-025-00256-8

**Published:** 2025-08-12

**Authors:** Yaoqi Huang, Haifeng Ye

**Affiliations:** Medical Center of Burn Plastic and Wound Repair, The First Affiliated Hospital; School of Basic Medical Sciences; Institute of Biomedical Innovation, Jiangxi Medical College, Nanchang University, Nanchang, 330006 China

**Keywords:** Female germline stem cells, Premature ovarian insufficiency, Artificial ovary, Ovarian repair

## Abstract

In the field of reproductive medicine, delaying ovarian aging and preserving fertility in cancer patients have long been core issues and relentless pursuits. Female germline stem cells (FGSCs) have been shown to repair aging or damaged ovarian structures and to restore ovarian reproductive and endocrine function. With their unlimited proliferation and directed differentiation into oocytes, FGSCs bring new hope to patients with ovarian insufficiency, malignant tumors, and others needing fertility preservation. In this review, we introduce the role of FGSCs in ovarian fertility preservation and regenerative repair, emphasizing the regulatory pathways of FGSCs in restoring ovarian function. We discuss the unique advantages of FGSCs in infertility treatment, including fertility preservation, animal gene editing, and regenerative medicine. This article aims to offer new research insights for advancing the clinical translation of FGSCs by exploring them from multiple perspectives, such as origin, regulation, and application.

## Background

Ovarian aging (age-related decline in ovarian function) and infertility issues resulting from ovarian diseases have become significant challenges in the field of global reproductive health (Touraine et al. 2024). Primary ovarian insufficiency (POI) is characterized by a reduced follicle count and a decline in oocyte quality, leading to an irreversible decrease in ovarian function and estrogen levels in women younger than 40 (Le et al. 2024). In recent years, the incidence of POI has risen significantly, with a global prevalence of 3.7%. In China, the prevalence has increased from 2.5% two decades ago to 15%, with ovarian-related factors accounting for 15% to 25% of cases (Federici et al. 2024; Hong et al. 2022). POI not only results in amenorrhea and infertility, but is also closely associated with systemic health issues such as osteoporosis, cardiovascular diseases, and cognitive impairments (Jiang et al. 2024; Theodorou et al. 2024). The mechanisms through which ovarian diseases affect fertility are complex. For example, ovarian cysts larger than 5 cm and cystectomy have been shown to significantly impact ovarian reserve six months after surgery (Bareghamyan et al. 2024; Shandley et al. 2023). Chemotherapeutic agents, especially alkylating agents, can directly damage follicular structures, leading to irreversible ovarian injury (Pu et al. 2023). Radiotherapy, bone marrow transplantation, and autoimmune disorders (e.g., lupus) trigger at least one million annual POI cases globally (McGlacken-Byrne and Conway 2022). In recent years, various intervention strategies have been proposed to address these issues. Ovarian tissue cryopreservation combined with in vitro activation (IVA) has been successfully applied in patients with primary ovarian insufficiency (POI) who have only a few remaining primordial follicles (Lee and Chang 2019), and healthy pregnancies and deliveries have been reported. GnRH-a may alleviate chemotherapy-induced damage to some extent (Blumenfeld and Evron 2015; Méndez et al. 2022). In addition, lifestyle interventions (e.g., antioxidant diet, BMI control) and early fertility preservation (e.g., egg freezing) have been shown to delay ovarian decline (Mintziori et al. 2019; Shelling and Ahmed Nasef 2023). However, how to remodel the impaired ovarian reproductive and endocrine functions has not been addressed. Therefore, prevention and treatment strategies for ovarian aging and chemotherapy-related infertility need to be strengthened.

## Evidence for the existence of female germline stem cells in the ovary

### Controversy over female germline stem cells

The question of whether FGSCs exist in mammals and differentiate into functional oocytes has been debated since the nineteenth century. Extensive studies have demonstrated that the total ovarian follicle pool is determined during the perinatal period, and no self-renewing stem cells or newly formed oocytes are present in the postnatal mouse ovary. The classical theory that “the mammalian ovarian follicle pool is fixed at birth” dominated the field for nearly 40 years (Borum 1961; Green et al. 1951, 1954; Horan and Williams 2017; Peters et al. 1962). However, since the discovery of FGSCs, accumulating evidence has challenged the century-old “fixed follicle pool theory” in reproductive medicine.

In 2004, Johnson et al. first suggested that proliferative germ cells exist in the postnatal mammalian ovary and contribute to follicular and oocyte regeneration (Johnson et al. 2004). By quantifying atretic and non-atretic follicles in C57BL/6 mice, they observed a rapid acceleration of follicular atresia after postnatal day 30, with only one-third of the original follicle pool remaining by day 42. Based on the atresia rate, they predicted complete depletion of the follicle pool within weeks. However, the total number of atretic follicles exceeded the actual loss, suggesting ongoing oocyte regeneration. Furthermore, transplanting wild-type ovaries into transgenic female mice ubiquitously expressing green fluorescent protein (GFP) revealed GFP-positive germ cells infiltrating host ovaries and forming new follicles. This finding contradicted the traditional view that the ovary contains only 1%~33% immature follicles at any stage, leading to the groundbreaking “oocyte regeneration theory.” Their subsequent research indicated that bone marrow (BM) expresses germline markers and restores oocyte production in sterilized mice via transplantation. Donor-derived oocytes, confirmed by morphology and molecular markers, suggest BM is a potential germ cell source sustaining oogenesis in adulthood (Johnson et al. 2005). Critics of Johnson’s theory (Gosden 2004) argued that their comparative counting method for atretic and non-atretic follicles had limitations. The criteria for identifying atretic follicles were deemed subjective and strain-specific (Faddy et al. 1983), and potential artifacts during tissue fixation might have overestimated atresia rates. Recent studies using single-cell RNA sequencing (scRNA-seq) and fluorescence-activated cell sorting (FACS) have failed to detect FGSCs in adult ovarian cortex samples (Wagner et al. 2020). However, Woods and Tilly (2023) criticized these findings, highlighting major flaws in Wagner et al.’s workflow. They noted the omission of cells with gene expression profiles identical to FGSCs, extensive cell damage/death during sample preparation, and the requirement for high cell viability in advanced techniques like scRNA-seq. Thus, the absence of FGSCs in these studies remains inconclusive, and mounting evidence continues to support the existence of FGSCs.

The controversy surrounding FGSCs primarily centers on the supporting faction's evidence from mouse models, where DDX4-positive cells generate oocytes, in contrast to opposing arguments that human ovaries lack active germline stem cell niches. This challenges the rationale for extrapolating rodent findings to humans. However, with FGSCs increasingly identified and cultured across diverse species alongside continuous advancements in research technologies, this debate will ultimately achieve more comprehensive resolution.

### Existence of female germline stem cells

In 2008, Bukovsky et al. (2008) isolated a small amount of adult ovarian surface tissue and cultured it in vitro for 5~6 days, observing the formation of follicle-like structures that extruded polar bodies and exhibited characteristics of secondary follicles. However, they isolated ovarian surface epithelial cells or ovarian stem cells (OSE), not specific FGSCs. As described in their study, the isolated OSE may differentiate into various cell types including mesenchymal, epithelial, granulosa, neural type cells, and oocytes. Zou et al. (2009) were the first to successfully isolate and purify FGSCs, demonstrating distinct germline stem cell properties. Subsequently, White, Lu, and Zhang independently isolated germline stem cells from ovaries of different species utilizing different approaches stem cells function (Lu et al. 2016; White et al. 2012; Zhang and Wu 2016). Addressing skepticism, recent findings (Alberico et al. 2022) demonstrate that optimized single-cell RNA sequencing (scRNA-seq) workflows can identify rare germline cells in adult ovarian cortical tissues that match ovarian stem cells (OSCs) but differ from other cell types, including oocytes and perivascular cells (PVCs). Further analysis revealed key molecular evidence of these germline cells initiating the first phase of meiosis within mature ovarian tissues.

To date, researchers have consistently identified ovarian germline stem cells (FGSCs) in the ovaries of rats, pigs, sheep, and humans, indicating their widespread presence across mammals (Li et al. 2022; Martin et al. 2019; Meng et al. 2023; Saber et al. 2022). Current challenges lie in comprehensively characterizing cell populations within adult mammalian ovaries and defining conditions that may support in vitro and in vivo neooogenesis.

### Characteristics of female germline stem cells

**Fig. 1:**
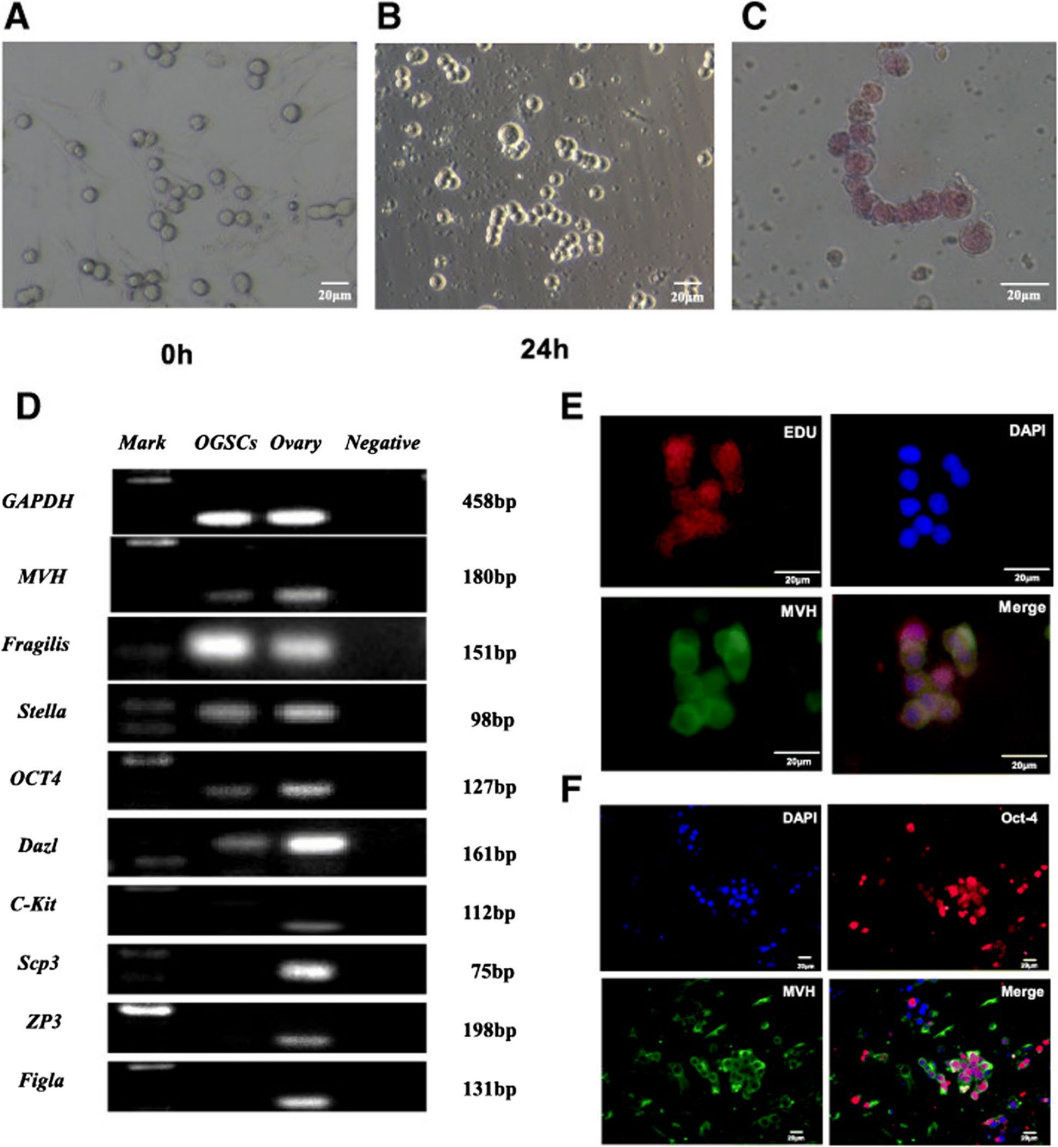
Identification of female germline stem cells. (**A**-**F **reproduced with the permission from Fig. 5 A-; Reproductive Biology and Endocrinology; https://doi.org/10.1186/s12958-021-00699-z). **A**, **B** The morphology of FGSCs; **C** Alkaline phosphatase staining; **D** Reverse transcription polymerase chain reaction (RT-PCR); **E**, **F** Immunofluorescence

FGSCs not only preserve the follicular pool but also exhibit characteristics typical of adult stem cells. Key characteristics of FGSCs are summarized as follows. Refer to Fig. 1.

1. Morphology and Growth Patterns: FGSCs resemble spermatogonial stem cells (SSCs), displaying a spherical shape, grape-like clusters, large and bright spherical nuclei, and distinct boundaries between nuclei and cytoplasm (Xie et al. 2014).
2. Germline-Specific Markers: FGSCs express germ cell-specific markers such as MVH, Dazl, OCT4, and Fragilis, aligning with the molecular profile of SSCs (Huang et al. 2021; Wang et al. 2023a, b; Zheng et al. 2023).
3. Proliferative Capacity: Dual immunofluorescence staining for MVH and BrdU confirmed that FGSCs retain mitotic activity while preserving germline identity (Xie et al. 2014).
4. Cell Cycle Regulation: FGSCs express cell cycle-related transcription factors (e.g., c-MYC, EGR-1) alongside high telomerase activity, in addition to TERT and alkaline phosphatase (White et al. 2012; Xie et al. 2014; Zou et al. 2009).
5. Differentiation Potential: Under estrogen supplementation, FGSCs differentiate into oocytes in vitro and generate fertile offspring upon transplantation into mouse ovaries (Wang et al. 2014, 2023a, b; Zou et al. 2009).

## Regulation of female germline stem cells

**Fig. 2:**
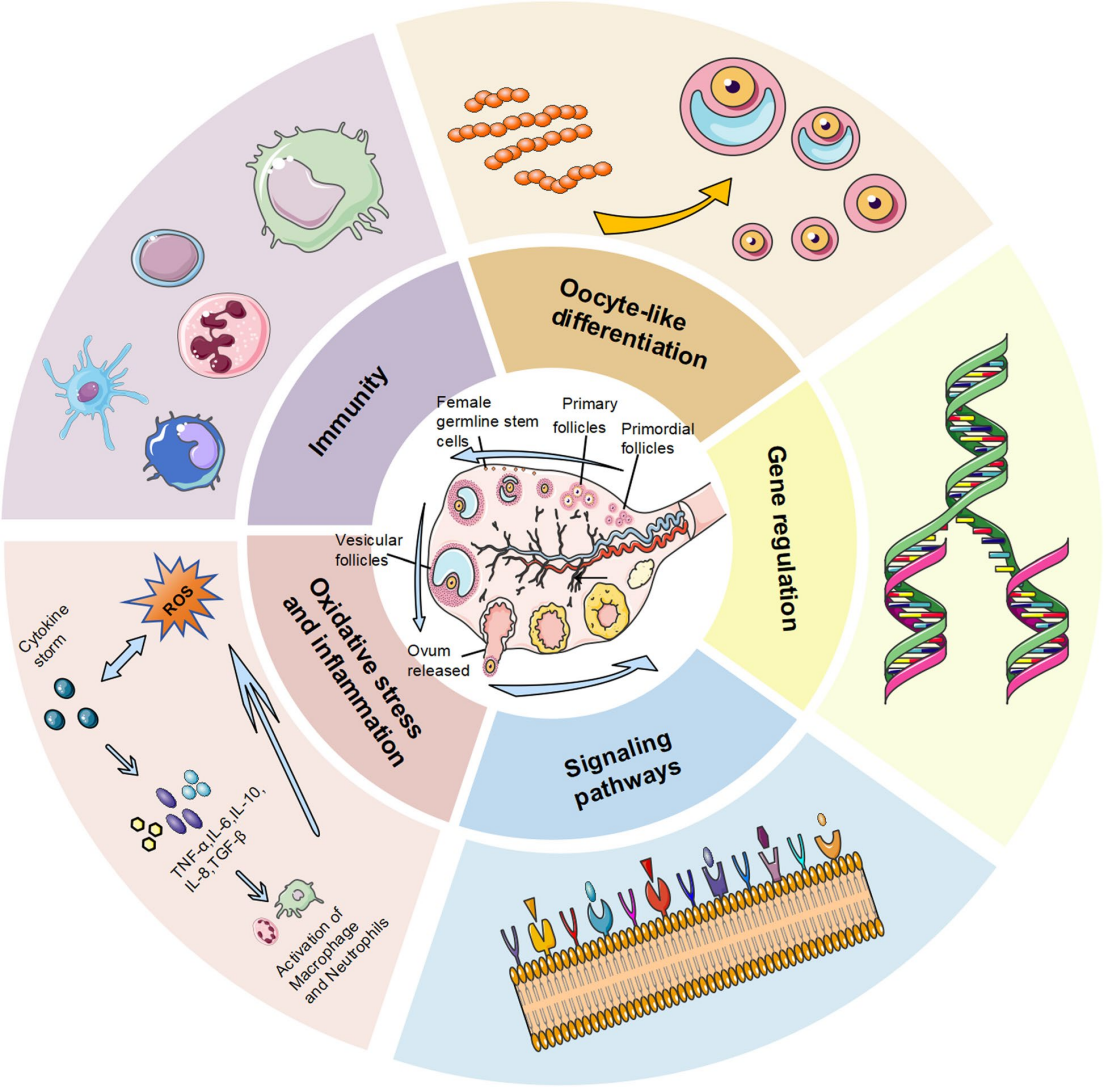
Pathways for FGSCs to restore ovarian function. (1) Immune Cell Mediated: Immune cells and their secreted factors participate in and promote the proliferation and differentiation of ovarian germline stem cells (OGSCs), thereby restoring ovarian endocrine function. (2) Direct Differentiation: FGSCs directly differentiate into primary oocytes, which further develop into mature follicles, replenishing the follicular pool. (3) Reduction of Stress & Inflammation: Reducing oxidative stress and inflammation restores ovarian function and FGSC activity, promoting FGSC survival. (4) Gene regulation: Upon estrogen (E2) stimulation, ERα binds to the Stra8 gene promoter (which is activated by retinoic acid) to induce Stra8 transcription, ultimately triggering oocyte development. (5) Regulation of signaling pathways: Modulating signaling pathways induces an autophagic protective response in FGSCs, ameliorating cellular senescence

FGSCs maintain ovarian reserve and functional homeostasis through self-renewal and directed differentiation. FGSCs promote follicular regeneration by activating signaling pathways such as Notch and Hedgehog (Hh), while secreting antioxidant factors (e.g., SOD, GPx) to counteract oxidative stress-induced damage to oocytes. FGSCs regulate ovarian function through multiple pathways (Fig. 2), with key mechanisms outlined below.

### Signaling pathways

**Fig. 3:**
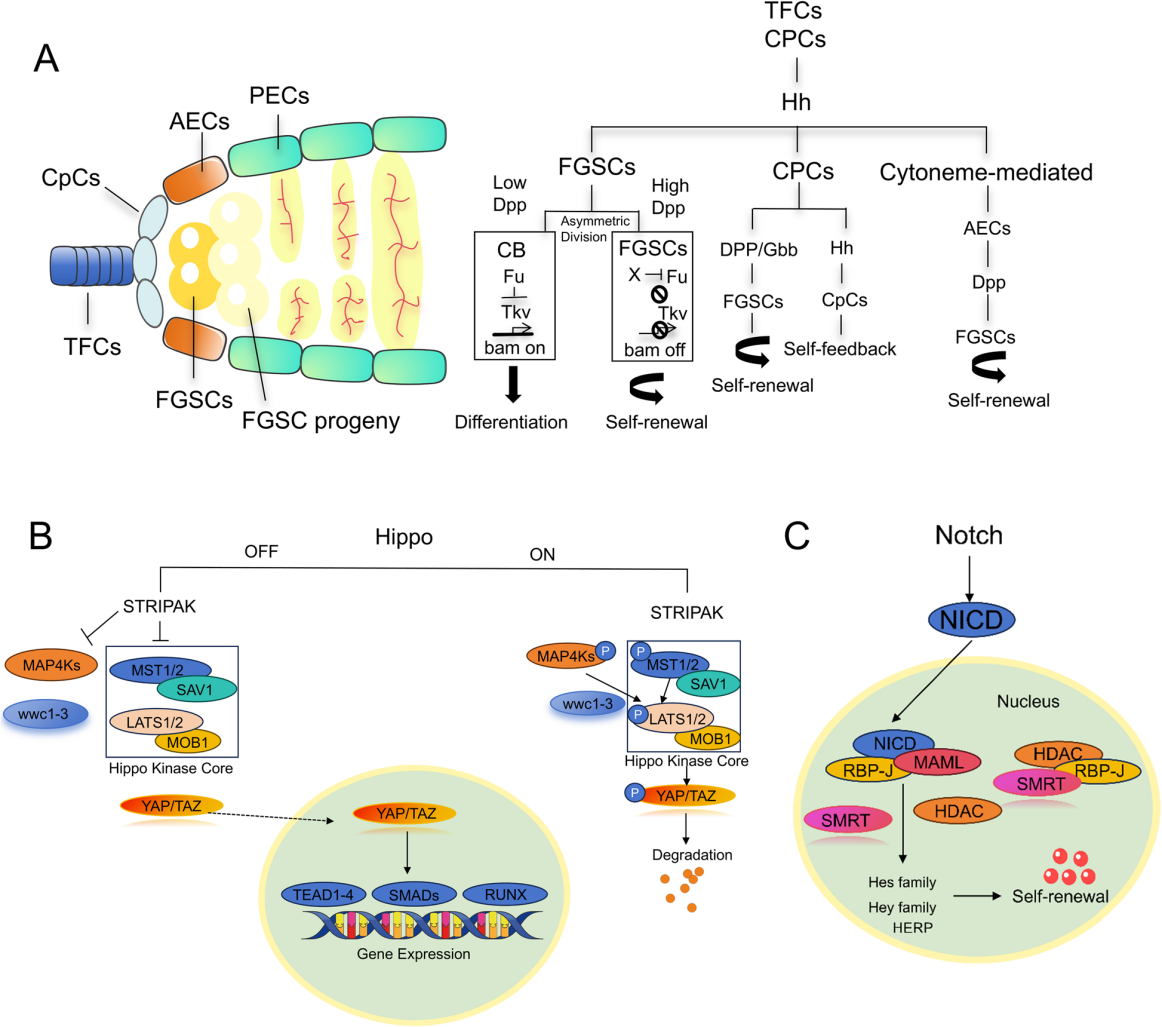
Schematic diagram of the regulatory mechanisms of the Hedgehog/Notch/Hippo signaling pathways. **A** Hh secreted by TFCs and CPCs can directly promote FGSC self-renewal, and can also secrete DPP/Gbb to indirectly promote FGSC self-renewal. **B** The STRIPAK complex acts upstream of kinases MAP4Ks and MST1/2, and inhibits the Hippo pathway. MAP4Ks or MST1/2 along with their scaffold protein SAV1 can phosphorylate LATS1/2 and its scaffold MOB1 with the assistance of WWC1-3. **C** When Notch ligands are present, they bind to Notch receptors on adjacent cells, forming the Notch intracellular domain (NICD). NICD enters the nucleus, interacts with CBF-1/RBP-Jκ, displaces SMRT and HDAC enzymes, and recruits MAML, thereby activating downstream genes and promoting cell proliferation

Several signaling pathways regulate FGSCs, thereby improving ovarian function (Fig. 3). The Hedgehog (Hh) pathway directly acts on FGSCs by activating downstream target genes to suppress differentiation and sustain self-renewal. It also indirectly regulates FGSCs through microenvironmental cascades. For instance, CpCs (cap cells) activate Hh signaling to induce expression of BMP family members Dpp and Gbb in anterior epithelial cells (AECs), while directly promoting Dpp transcription in CpCs themselves (Li et al. 2017). Elevated BMP (Dpp) signaling inhibits the differentiation factor Bam, further blocking FGSCs differentiation (Inaba et al. 2016; Rojas-Ríos et al. 2012). FGSCs differentiation is dynamically balanced: during differentiation initiation, the Hh pathway’s positive regulator Fused (Fu) is upregulated. Fu promotes FGSCs transformation into mature germ cells by mediating ubiquitin-dependent degradation of the BMP receptor Tkv, thereby attenuating BMP-mediated differentiation suppression (Lu et al. 2012; Xia et al. 2010, 2012).

The Notch pathway plays critical roles in cell proliferation, differentiation, and fate determination, significantly influencing both somatic and germline stem cells. Notch regulates FGSCs stemness maintenance and differentiation via intercellular communication (Hsu et al. 2019). In Drosophila, ovarian stem cell niche formation relies on Notch signaling. Germline stem cells (GSCs) reside in somatic niches where Notch is pivotal: activating Delta in germ cells or Notch in somatic cells increases niche cell numbers, inducing additional GSC formation. Conversely, GSCs lacking functional Notch ligands (Delta/Serrate) fail to activate TGF-beta signaling, leading to differentiation and niche exit (Ward et al. 2006). Functionally, Notch signaling decline correlates with ovarian aging, as reduced Notch activity causes FGSCs depletion and excessive primordial follicle atresia (Pan et al. 2015; Song et al. 2007).

The Hippo pathway critically regulates ovarian function, particularly FGSCs. Through kinase cascades, Hippo controls cell proliferation, apoptosis, and organ size (Fu et al. 2024). Hippo activation phosphorylates downstream coactivators YAP and TAZ, retaining them in the cytoplasm for degradation, thereby inhibiting proliferation and promoting apoptosis (Kwon et al. 2013; Yin et al. 2013). Conversely, Hippo inhibition allows dephosphorylated YAP/TAZ to translocate into the nucleus, binding TEAD family transcription factors to activate genes driving proliferation and stem cell renewal (Pocaterra et al. 2020). In ovaries, Hippo signaling is essential for FGSCs homeostasis. Dysregulated Hippo signaling is linked to ovarian aging and disease, offering insights into pathology and therapeutic strategies (Clark et al. 2022). In mouse ovarian cortex, Hippo components (LATS2, MST1) and germline markers (MVH, OCT4) co-express. Their levels and colocalization decline with age, while YAP1 is prevalent in 2-month-old mice but absent in 20-month-old mice, suggesting Hippo’s role in FGSCs dynamics during physiological and pathological ovarian aging (Li et al. 2015).

### Stem cell niche

Schofield’s (1978 ) stem cell niche hypothesis posits that niches comprise extracellular matrix, niche cells, granulocytes, blood vessels, immune cells, and secreted factors. Growing evidence implicates FGSCs niche dysfunction as a key driver of ovarian failure, potentially more impactful than FGSCs aging itself (Hong et al. 2022). However, inflammatory microenvironments disrupt niche integrity (Dooley et al. 2014). Macrophages, with their heterogeneity and cytokine-secreting adaptability, may stabilize niches by clearing senescent red blood cells and necrotic tissue via IL-10 and TNF-α (Casanova-Acebes et al. 2014).

### Oxidative stress and chronic inflammation

Oxidative stress and chronic inflammation accelerate stem cell aging. Follicular hypoxia drives ovarian aging by reducing intracellular pH and oocyte metabolism, destabilizing meiotic spindles (Molinari et al. 2016). Hypoxia activates oxygen sensor HIF-1α, enhancing glycolysis, angiogenesis, leukocyte migration, inflammation, and follicular wall rupture (Kim et al. 2009; Neeman et al. 1997). It also induces ROS overproduction, exacerbating oxidative ovarian damage (McGarry et al. 2018). ROS and inflammation form a vicious cycle: ROS activate NLRP3 and NF-κB, upregulating IL-1β, IL-6, and TNF-α, which further amplify oxidative stress and aging (Atalay Ekiner et al. 2024; McGarry et al. 2018; Tucker et al. 2015; Zhang et al. 2024). Antioxidant interventions like resveratrol (RES) restore ovarian function and FGSCs viability in POI models by reducing oxidative stress and inflammation (Breuss et al. 2019; Nadile et al. 2022; Pyo et al. 2020; Ren et al. 2021; Zhou et al. 2021; Jiang et al. 2019). Chitosan oligosaccharides improve ovarian microenvironments and stimulate FGSCs proliferation via immune-related factors (Huang et al. 2021; Li et al. 2023). Spermidine (SPD) protects FGSCs from H2O2-induced senescence by inducing autophagy via PI3K/Akt (Yuan et al. 2021). These strategies may delay reproductive aging and preserve fertility.

### Others

Polycystic Ovary Syndrome (PCOS), a common endocrine disorder, is linked to hyperandrogenism, glucose intolerance, and cystic ovaries. Metformin, a first-line antidiabetic drug, significantly increases proliferative FGSCs and upregulates PCNA, cyclin D2, p-mTOR, and p-AMPK in PCOS mice, restoring FGSCs function via AMPK/mTOR signaling (Wang et al. 2022). Soy isoflavones activate Akt signaling by upregulating Clec11a, promoting FGSCs survival and proliferation (Li et al. 2022).

These findings, along with transplantation and genetic lineage-tracing data, confirm FGSCs’ capacity to generate healthy oocytes, embryos, and offspring, underscoring their potential in managing female fertility and ovarian disorders.

## Clinical applications and translational prospects of female germline stem cells

### Fertility preservation

FGSCs present innovative strategies for fertility preservation and the delay of menopause. FGSCs can either be transplanted into ovaries to initiate oogenesis for producing fertile offspring or cultured in vitro and differentiated into oocytes after injection into human ovarian cortical tissue, followed by xenotransplantation into immunodeficient adult female mice (Cheng et al. 2022; Ding et al. 2016a; Zou et al. 2009). Functional oocytes can also be derived in vitro from spermatogonial stem cells (SSCs) (Luo et al. 2021).

White et al. isolated FGSCs from adult human ovaries and transplanted them into mouse ovarian cortices, observing immature oocytes and granulosa cell formation (White et al. 2012). Satirapod’s team confirmed that mouse FGSCs express estrogen receptor-α (ERα), which interacts with retinoic acid-induced Stra8 during oogenesis to drive Stra8 expression (Satirapod et al. 2020). Xiong et al. restored fertility in chemotherapy-induced infertile mice by transplanting in vitro-cultured FGSCs into their ovaries (Xiong et al. 2015). Wu et al. (2017) showed that FGSCs initiate differentiation into early oocytes upon reaching the ovarian cortex, with transplanted FGSCs (F-TFs) restoring ovarian function and producing offspring. Collectively, these findings confirm the existence of proliferative germ cells in postnatal mammalian ovaries, sustaining oocyte and follicle production.

Bukovsky and Presl (1979) hypothesized immune system-ovary interactions in regulating reproduction and reproductive disorders (Bukovsky and Presl 1979). Subsequent studies support this: athymic (nude) female mice exhibit reduced gonadotropin levels, reversible via neonatal thymosin therapy (Goya et al. 2007). In humans, ovarian surface epithelial cells (OSCs) act as bipotent stem cells generating germ and granulosa cells. Immune regulation participates in physiological neo-oogenesis and follicular renewal during fetal and reproductive stages, with FGSCS proliferation and differentiation modulated by immune cells and their secreted factors (Bukovsky and Caudle 2012; Ye et al. 2016).

### Organoids and in vitro models

Wu et al. (Li X et al. 2021) established ovarian organoids from mouse FGSCs using a 3D culture system. These organoids, resembling normal ovaries, contained follicles and secreted hormones. Single-cell sequencing identified six cell populations, including germ, granulosa, and theca cells, with distinct gene expression profiles. Follicles from organoids matured in vitro and produced normal offspring via fertilization. Another team used whole-organ decellularization to create 3D bioscaffolds mimicking the natural ovarian microenvironment, preserving native microarchitecture and biochemical cues. FGSCs purified via MACS adhered to these scaffolds within 24 h, colonized the extracellular matrix (ECM), and formed cluster-like structures (Pennarossa et al. 2021). Luo et al. (Luo et al. 2023) developed semiconductor polymer dot (Pdot)-based siRNA nanocomposites for targeted gene knockdown in FGSCs. Pdots exhibited high fluorescence, enabling real-time tracking of cellular uptake, intracellular trafficking, and exocytosis. Notably, Pdots showed minimal cytotoxicity and differentiation interference, escaping lysosomes for efficient extracellular secretion, making them ideal nanocarriers. Pdot-siRNA also penetrated FGSC-derived 3D ovarian organoids, effectively downregulating target genes.

**Fig. 4:**
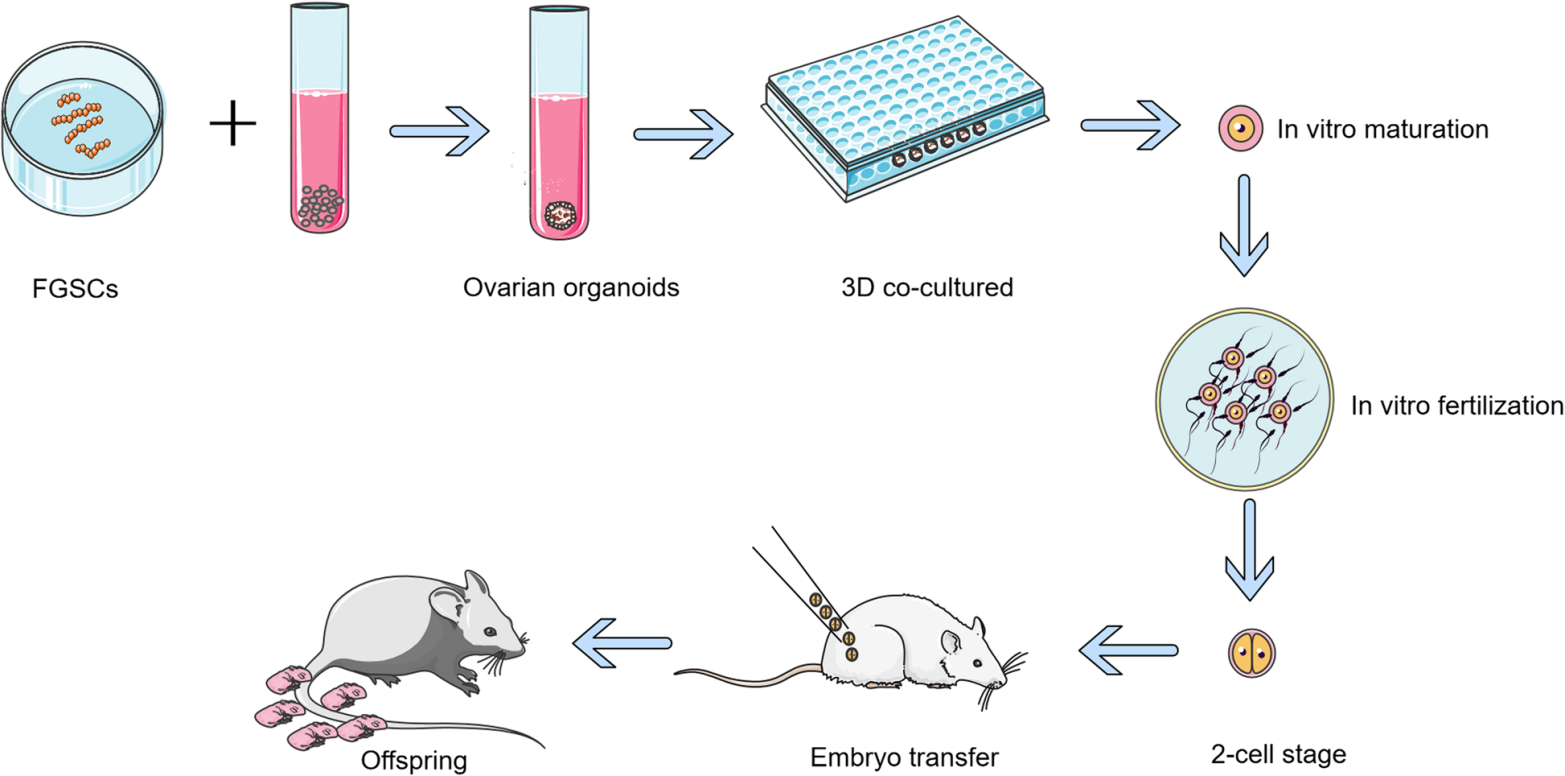
Schematic diagram of ovarian germline stem cell (FGSC)-derived ovary-like organoid generation. FGSCs were combined with 3D bioscaffolds and co-cultured in 96-well plates. The culture medium was modified under varying conditions, supplemented with growth factors, until ovary-like organoids containing oocyte-like follicles were observed. Oocytes isolated from these organoids were used for embryo transfer via assisted reproductive technologies (ART) to generate offspring in mice

Thus, bioengineered ovarian prosthetics (Fig. 4) using FGSCs represent an innovative strategy for safe fertility restoration, particularly in cancer survivors.

### Stem cell and gene therapy

FGSCs are increasingly recognized for their potential in treating infertility, preserving fertility, enabling animal gene editing, and advancing regenerative medicine. Ovarian dysfunction or insufficiency, caused by genetic disorders, autoimmunity, or injury (Chon et al. 2021; Ishizuka 2021; Szeliga et al. 2021), as well as chemotherapy/radiation-induced ovarian failure (Duffy et al. and Allen 2009; Jadoul et al. 2010; Park et al. 2024), are major contributors to infertility. Current methods (oocyte/embryo cryopreservation, ovarian tissue freezing) risk reintroducing malignant cells in cancer survivors (Yan et al. 2022). Stem cell therapies, including embryonic stem cells (ESCs), mesenchymal stem cells (MSCs), and induced pluripotent stem cells (iPSCs), are emerging as promising alternatives (Sadeghi et al. 2024). FGSCs, with their capacities for self-renewal and differentiation, hold unique potential for the treatment of ovarian diseases.

FGSCs’ innate ability to differentiate into oocytes makes them ideal for generating transgenic animals, circumventing costly and labor-intensive methods like pronuclear DNA injection or somatic cell nuclear transfer (Brinster and Zimmermann 1994; Pursel et al. 1989; Wilmut et al. 1991). Zhang et al. (2011) achieved transgenic offspring within two months by introducing functional genes or knocking down targets in FGSCs isolated from neonatal and adult mice, with efficiency (29%~37%) far exceeding SSCs (4.5%) (Nagano et al. 2001). This approach is broadly applicable to mammals, including livestock, advancing gene therapy and biotechnology.

FGSCs demonstrate versatile applications: Ovarian Tissue Engineering: Co-cultured with 3D bioscaffolds, they enable artificial ovary models to restore ovarian function. Oocyte Regeneration: Differentiating into oocytes in vitro*/*in vivo, they replenish follicular pools, offering strategies for POI and ovarian tissue regeneration. Transgenic Models: Used to create gene-edited animals for studying reproduction and gene function. Assisted Reproduction: Potential new avenues for women with declining fertility. Despite challenges, FGSC-based therapies show significant potential for fertility preservation in ovarian damage or cancer patients.

Reconstructing human ovary-like microenvironments and elucidating signaling pathways for in vitro oogenesis and folliculogenesis will enhance FGSCs differentiation efficiency, offering new hope for female fertility preservation.

## Limitations of ovarian germline stem cell research

### Technical bottlenecks of FGSCs

The isolation and identification of ovarian germline stem cells (FGSCs) remain challenging. FGSCs lack highly specific surface markers, and current isolation methods frequently contaminate samples with other ovarian cells, compromising purity. Clinically, FGSC isolation requires ovarian tissue extraction—a process constrained by limited sample availability, scarcity of sources, and invasive risks. In humans, FGSCs are obtained from ovarian cortical tissue within follicular aspirates (Ding et al. 2016a, b). However, low ovarian tissue yield in aspirates leads to inconsistent success rates in establishing cell lines.

FGSCs exhibit low efficiency in differentiating into mature oocytes in vitro and frequently undergo senescence or lose differentiation potential during expansion, resulting in significant depletion of stemness after passaging. Traditional 2-dimensional (2D) culture systems fail to adequately simulate the ovarian microenvironment in vitro. Compared to mesenchymal stem cells, long-term culture protocols for FGSCs remain unoptimized. While Matrigel-based 3D culture enhances cell viability and stemness maintenance, critical parameters—including Matrigel concentration, embedding methods (e.g., co-culture with feeder cells), and batch-to-batch consistency—require repeated optimization (Zhang et al. 2023).

### Clinical safety of FGSC therapy

Current evidence lacks long-term safety data for FGSC therapy, as it may induce tumor formation and retain epigenetic memory from source cells, necessitating consideration of potential clinical risks (Jahnukainen et al. 2015; Telfer 2019). While transplanted cells require integration into the host ovarian stroma, fibrotic or inflammatory microenvironments may inhibit FGSC engraftment. Additionally, allogeneic FGSCs could trigger host immune attacks.

Germline gene editing in human FGSCs demands extreme caution due to heritability to offspring. The ovarian microenvironment's complexity, coupled with stem cell therapy remaining in clinical trial/research phases, results in significant interindividual variability in treatment responses among patients (Li et al. 2021; Yan et al. 2020). Post-transplantation outcomes are likely influenced by patient age and underlying etiology.

### Solutions for research limitations of FGSCs

Optimization of culture and differentiation systems for FGSCs can be achieved by combining natural inducers (e.g., *Cistanche* polysaccharides) to target the TGF-β pathway, thereby enhancing differentiation synchrony (Qiu et al. 2022). Inhibiting EED activity effectively improves culture efficiency and cell yield (Wang et al. 2023a, b). Serum-free and feeder-free FGSC culture systems can be established using Matrigel optimization (Li X et al. 2022).

To address clinical safety concerns, multimodal imaging (e.g., MRI-fluorescence dual labeling) enables real-time monitoring of transplanted cell distribution in vivo for early detection of abnormal proliferation. Stratifying patients by etiology reduces heterogeneity in treatment outcomes. Long-term animal transplantation tracking (> 6 months) should implement mesenchymal stem cell safety assessment protocols (Dunn et al. 2021) to monitor teratoma formation and intergenerational reproductive effects.

Developing ovarian cell (granulosa/supporting cells)-FGSC co-culture organoids mimics physiological follicular microenvironments, advancing differentiation mechanism research (Wang et al. 2023a, b). Additionally, 3D bioscaffolds replicating natural ovarian niches can be engineered. Stimulated purified FGSCs may repopulate decellularized ovarian scaffolds to restore function (Pennarossa et al. 2021). These technologies represent promising solutions for investigating ovarian reproductive and endocrine functions.

## Conclusions and perspectives

Preserving fertility remains a pivotal focus in medicine, enabling women to retain reproductive health. Stem cells in adult ovaries hold significant implications for reproductive medicine. Current fertility preservation techniques include embryo cryopreservation, oocyte cryopreservation, and ovarian tissue cryopreservation. While embryo and oocyte freezing are well-established, ovarian tissue cryopreservation—requiring surgical removal, freezing, and subsequent transplantation of thawed tissue—is still in its early clinical stages (Anderson et al. 2020; ASRM 2019). Advances in cancer treatment have improved survival rates, yet surgery, chemotherapy, and radiotherapy often severely damage ovarian function. Adolescent or reproductive-age female patients achieving long-term survival after cancer remission or cure face diminished or lost fertility. Internationally, ovarian tissue cryopreservation and transplantation (OTCT) is recognized as the sole method to preserve ovarian function and fertility in children and women who cannot delay cancer therapies. OTCT also represents the most effective and promising approach for fertility preservation. Years later, thawed autologous ovarian tissue can be transplanted back to restore ovarian function and fertility. However, existing studies have demonstrated that dual damage from cryopreservation and ischemia leads to severe ovarian reserve depletion and mitochondrial energy metabolism dysfunction in oocytes (Wu et al. 2023). To address these challenges, the potential application of FGSCs in this field offers substantial promise for reproductive medicine researchers. On one hand, FGSCs can differentiate directionally into functional oocytes; on the other hand, they may be co-cultured with other cells or biomaterials, potentially enabling the generation of ovarian organoids (Zhang et al. 2023).

The ovary can be enzymatically dissociated into isolated cells or primordial follicles. When reaggregated and transplanted into recipient animals, these cells regenerate functional follicles capable of ovulation (Gosden 1990). During ovarian transplantation and tissue repair, germ and granulosa cells may reorganize between donor and host primordial follicles. Notably, somatic and germ cells isolated from mouse and rat ovaries can be recombined in vitro to reconstruct follicles, which are then transplanted into severe combined immunodeficient (SCID) mice (Eppig and Wigglesworth 2000). Thus, developing novel artificial ovarian models co-cultured with diverse ovarian cells to study functional restoration technologies is a critical challenge.

While germline stem cells like FGSCs are valued for their fertility-restoring potential, their limited accessibility and incomplete understanding of oocyte conversion mechanisms hinder therapeutic applications. Additionally, the tumorigenicity of stem cell therapies remains a concern. Similar to spermatogonial stem cells (SSCs) in male infertility treatments, FGSCs carry a residual risk of malignant transformation (Simon et al. 2010).

In summary, FGSCs offer novel strategies for treating ovarian disorders and advancing transgenic animal research or elite livestock breeding. Despite challenges, the scientific community and public remain optimistic about their potential to revolutionize ovarian disease management and infertility therapies.

## Data Availability

Not applicable.

## Abbreviations

FGSCs: Female germline stem cells
POI: Premature Ovarian Insufficiency
SSCs: Spermatogonial stem cells
FACS: Fluorescence-activated cell sorting
scRNA-seq: Single-cell RNA sequencing
DDX4: DEAD-box helicase 4
PVCs: Perivascular cells
OSE: Ovarian surface epithelium
GFP: Green fluorescent protein
ERα: Estrogen receptor-alpha
Stra8: Retinoic acid gene 8
ROS: Reactive oxygen species
PCOS: Polycystic Ovary Syndrome
Clec11a: C-type lectin domain family 11 member a
ESCs: Embryonic stem cells
MSCs: Mesenchymal stem cells
iPSCs: Induced pluripotent stem cells
MACS: Magnetic-activated cell sorting
Pdots: Semiconductor polymer microdroplets
